# Contrasting effects of reinforcer rate and magnitude on differential resistance to change in humans

**DOI:** 10.1002/jeab.70027

**Published:** 2025-06-30

**Authors:** Carlos Eduardo Costa, Karina Pinheiro da Silva, André Connor de Méo Luiz, André Marques Choinski, Kennon A. Lattal

**Affiliations:** ^1^ Universidade Estadual de Londrina, Londrina, Paraná Brasil; ^2^ Integral: Engenharia Comportamental, Londrina, Paraná Brasil; ^3^ Instituto Continuum, Londrina, Paraná Brasil; ^4^ Universidade Federal do Paraná, Curitiba, Paraná Brasil; ^5^ West Virginia University Morgantown WV USA

**Keywords:** humans, physical effort, reinforcement magnitude, reinforcement rate, resistance to change

## Abstract

The effects of the magnitude of reinforcement on the resistance to change of humans engaged in a computer task were examined in two experiments. In each, responding was disrupted by increasing the force requirement of the required response. In Experiment 1, the participants were exposed to a multiple variable‐interval (VI) VI schedule of reinforcement. Responses meeting the VI requirement resulted in the addition of a monetary value to the computer screen. At the end of each session, the monetary value was exchanged for real money. In Experiment 2, snacks or money provided at the time earned were the reinforcers. There was no differential resistance to change as a function of reinforcer magnitude in either experiment. These findings led to Experiment 3, in which the participants from Experiment 2 were exposed to schedules arranging different reinforcement rates using as reinforcers snacks, money at the time it was earned, and points exchangeable for money at the session's end. There was greater resistance to change in the VI component with a higher reinforcement rate. The results are discussed in relation to the varied effects of reinforcer magnitude on both response rates and resistance to change.

The efficacy of a reinforcer depends on its frequency, immediacy, and magnitude. Generally, reinforcers delivered more frequently and closer in time to the response that produces them maintain higher rates of responding than do those delivered less frequently and with longer delays. Reinforcer magnitude presents a more complicated relation with response rate (Bonem & Crossman, 1988). Part of this complexity is because different operations are used to define it (e.g., concentration of sucrose or duration of hopper access with laboratory animals and number of points earned with human participants). Even with a constant definition of magnitude, such as the duration of access to a food hopper, the effects may still vary as a function of the schedule arranging the reinforcers (e.g., a single versus a concurrent schedule, Catania, 1963) or the design of the hopper from which the grain reinforcer is delivered (Epstein, 1981).

Another index of reinforcer efficacy is the relative resistance to change of a response maintained by different parameters of the reinforcer when the disrupting event occurs. As with response rates, reinforcers delivered more frequently and immediately result in responding that is more resistant to change than those delivered less frequently or with longer delays (Craig et al., 2014; Greer et al., 2016; Nevin, 1974, 2015; Nevin & Wacker, 2013; Podlesnik & DeLeon, 2015). Although resistance to change has been investigated with humans in both basic and applied contexts, it has been studied most extensively as a function of reinforcement rate and delay (cf. Trump et al., 2021).

Reinforcer magnitude effects on resistance to change first were investigated by Nevin (1974, Experiment 3). He maintained key pecking of pigeons with a multiple variable‐interval (VI) 1‐min VI 1‐min schedule, arranging either 2.5 or 7.5 s of access to the food hopper in either component. Higher response rates were maintained by the longer duration reinforcer with one but not the other pigeon. Responding of both pigeons decreased systematically as the rate of response‐independent food delivery during the intercomponent interval increased. For the pigeon with differentiated responding between the two components, responding maintained by the longer duration reinforcer was more resistant to change. For the other pigeon, differences in resistance to change as a function of reinforcer duration were minimally higher in the component with the longer duration reinforcer. More reliably differential effects of disruptors on key‐pecking of pigeons maintained by different magnitudes of reinforcement were reported by Harper and McLean (1992) and Harper (1996). Responding maintained by longer as opposed to shorter duration food access was more resistant to disruption by response‐independent reinforcers delivered during the intercomponent interval.

Using a different procedure, McComas et al. (2008) investigated reinforcer magnitude effects on resistance to change of the operant responding of undergraduate students. The participants used a mouse to click on a computer‐screen button that resulted in points later exchangeable for money. VI 30‐s schedules were in effect in both components of a multiple schedule. In one of the multiple schedule components—associated with a yellow background stimulus—the two schedules concurrently in effect arranged either 8 points or 1 point as the consequence for responding on the respective concurrently available operanda. In the other multiple schedule component—associated with a green background stimulus—the two schedules concurrently in effect arranged either 2 points or 1 point as the consequence for responding on the respective concurrently available operanda. One of the four planned participants was dropped for nondifferential responding and failure to attain stable responding; of the remaining three, none responded consistently higher in the yellow component, where 9 points total (8 + 1) were arranged as reinforcers on the two operanda. Two of the three participants, however, showed greater persistence of responding during extinction in the yellow (richer, 9 points total) component. For the same two participants showing greater persistence in the multiple schedule component arranging a total of 9 points, responding during the VI associated with 1 point was more resistant to change when it operated concurrently with the VI schedule delivering 8 points than when that same schedule arranging 1‐point reinforcers operated concurrently with the VI schedule delivering 2 points. Overall, McComas et al.'s data are suggestive but not conclusive that there is a relation between persistence and resistance to change even when different magnitudes fail to differentially control response rates maintained by reinforcement schedules arranging nominally different reinforcement magnitudes. Given the novelty of McComas et al.'s procedure and the lack of consistent results, one impetus for the present experiment was the relative paucity of laboratory research on human resistance to change as a function of reinforcer magnitude in a multiple schedule.

A general limitation of the analysis of resistance to change is that it has been predominantly tested with both nonhuman and human animals using primarily only three procedures: extinction, the delivery of response‐independent food during the intercomponent interval (ICI) of a multiple schedule, and presession access to the reinforcer. Expanding the types of disruptors investigated, Costa et al. (2024) found that both point loss and increased response force requirements differentially suppressed responding of humans maintained by more or less frequent point delivery. Thus, a second impetus for the present experiment was to examine the disruption of human operant responding maintained by different reinforcement magnitudes as a function of the required force of such responding. Because the results of this examination revealed little difference between the resistance of responding maintained by different reinforcement magnitudes, a third purpose was to compare the resistance of the same participants' responding under different magnitudes of reinforcement to such resistance under different reinforcement rates.

## EXPERIMENT 1

Experiment 1 was designed to examine the effects of the magnitude of reinforcement on resistance to change of humans engaged in a computer task. The participants were exposed to a multiple VI VI schedule, and an increase in the response force required served as the disruptor.

### Method

#### Participants

The participants were three female undergraduate students (P1–P3), aged 17–20, without prior experimental histories. The invitation informed participants they would participate in a study about human behavior and spend approximately 30 min during each laboratory visit, 2–5 times per week. Participants were debriefed about the goals of the experiment at the end of the last session of the experiment. The Local Committee for Ethical Research approved all procedures performed with the participants (CAAE: 55235516.5.0000.0093, Protocol 1.537.824/2016).

#### Apparatus

Sessions were conducted in two 3‐m^2^ rooms containing a desk, a chair, a mouse, a spring‐loaded button (described below), and a computer. White noise was reproduced through headphones connected to the computer to mask extraneous sounds. The experimental task and recording of the participant's responses were made in the software ProgRef v4 (Becker & Costa, 2024). The *Stability Check* software (Costa & Cançado, 2012) calculated response‐rate stability.

The operanda were, in different phases of the experiment, either a spring‐loaded button or a standard computer mouse connected via USB to the computer. The spring‐loaded button was in a fixed position throughout the experiment. It consisted of a nylon box 13 (height) × 13 (length) × 13 cm (wide), with a 3.53‐cm (diameter) cylinder attached at the top. When pressed 3.5 cm down, the cylinder activated a button that registered the press as a left mouse click on a computer. The steel spring inside the button could be changed to require different forces to click the button (see Lacerda et al., 2023). Response force requirements were calculated according to Hooke's law (Aranha et al., 2016). The force requirement for the mouse to record a response was approximately 1 N, and it was 70 N for the spring‐loaded button.

#### Procedure

Before the first session, the participants read and signed an informed consent form describing the number and duration of sessions. The task consisted of accumulating a monetary value displayed on the computer screen, which participants would then receive in real currency at the end of each session. The participants were then asked to leave all personal belongings (e.g., bags, watches, and cell phones) outside the experimental room.

The computer screen layout in either component consisted of a gray background with a 10.0‐ × 2.0‐cm response button in the screen's lower center and a 4.5‐ × 0.5‐cm consummatory response button in the upper right corner of the screen. The color of the response button changed depending on the component of the multiple schedule. Above the response button, an 8.0‐ × 2.9‐cm counter (blue on a black background) displayed the value in currency used in Brazil (R$) earned in each session.

Once a response met the contingency, an image of a smiley face appeared below the consummatory response button, signaling the availability of one reinforcer. The smiley face remained on the screen until the participant clicked the consummatory response button (baseline phase) or pressed the Escape key on the keyboard (test phase) to add monetary value to the counter. At the end of each session, the screen displayed the total value earned and the message “Call the Experimenter.” The participants were paid according to their performance at the end of each session based on the value displayed on the counter. Table 1 shows the phases of Experiment 1 for each participant, the multiple schedule used, the reinforcer value for each multiple schedule component, the reinforcement ratio obtained between the components, the operandum used, and the number of sessions in each phase (in parentheses). The reinforcement ratio involved dividing the mean number of reinforcements obtained in the component with the lower magnitude of reinforcement (lean) by the mean number of reinforcements obtained in the component with the higher magnitude (rich) during all sessions of each phase. Values close to 1.0 indicate that the number of reinforcers obtained was identical in both components; values greater than 1 indicate a greater quantity of reinforcements in the lean component, whereas values less than 1 indicate a greater number of reinforcers obtained in the rich component.

**TABLE 1:** Procedure for Experiment 1.

	Participants		
Phases	P1	P2	P3
TR	VI 10 s / VI 10 s	VI 10 s / VI 10 s	VI 10 s / VI 10 s
	R$0.02 / R$0.20	R$0.02 / R$0.20	R$0.02 / R$0.20
	1.00	0.89	0.91
	Mouse 1 N	Mouse 1 N	Mouse 1 N
	(1)	(1)	(1)
	VI 20 s VI 20 s	VI 20 s VI 20 s	VI 20 s VI 20 s
	R$0.03 / R$0.30	R$0.03 / R$0.30	R$0.03 / R$0.30
	1.04	1.00	1.00
	Mouse 1 N	Mouse 1 N	Mouse 1 N
	(1)	(1)	(1)
BL1	VI 30 s VI 30 s	VI 30 s VI 30 s	VI 30 s VI 30 s
	R$0.05 / R$0.50	R$0.05 / R$0.50	R$0.05 / R$0.50
	1.00	1.01	1.01
	Mouse 1 N	Mouse 1 N	Mouse 1 N
	(4)	(4)	(6)
T1	VI 30 s VI 30 s	VI 30 s VI 30 s	VI 30 s VI 30 s
	R$0.05 / R$0.50	R$0.05 / R$0.50	R$0.05 / R$0.50
	1.00	1.01	0.97
	Spring 70 N	Spring 70 N	Spring 70 N
	(4)	(10)	(10)
BL2	VI 30 s VI 30 s	VI 30 s VI 30 s	VI 30 s VI 30 s
	R$0.05 / R$0.50	R$0.05 / R$0.50	R$0.05 / R$0.50
	1.00	0.98	1.01
	Mouse 1 N	Mouse 1 N	Mouse 1 N
	(4)	(4)	(4)
T2	VI 30 s VI 30 s	VI 30 s VI 30 s	VI 30 s VI 30 s
	R$0.05 / R$0.50	R$0.05 / R$0.50	R$0.05 / R$0.50
	1.00	0.99	0.95
	Spring 70 N	Spring 70 N	Spring 70 N
	(10)	(5)	(10)

*Note*: TR = training; BL = baseline; T = test; N = Newtons. The monetary value shown on the screen was in Brazilian currency (R$). At the time the experiment was conducted, R$0.05 was approximately equivalent to US$0.015. The number below the monetary values indicates the reinforcement ratio obtained between components of the multiple schedule (more details in the text).

#### Training

Participants were asked to read the following instructions (in Portuguese). The parts of the text in italics changed before initiating the test sessions. However, there were no italics in the instructions presented to the participants.

This study is not about intelligence or personality. *To start the session*, *click the mouse cursor on the button that will appear at the bottom center of the screen [Start Session]. By clicking the mouse cursor on the response button (green or red central button)*, *a smiley face will eventually appear in the upper right corner of the screen. When this happens*, *click on the button in the upper right corner of the monitor (above the smiley face)*, *and a monetary value will be added in the window in the screen's upper center*, *above the response button (green or red). The monetary value will not be added if you do not press this button above the smiley face*. The experimenter is not authorized to give any additional information. Good work!

Participants were exposed to two 16‐min sessions under a multiple VI 10‐s VI 10‐s (first session) and VI 20‐s VI 20‐s (second session) schedule of reinforcement (excluding the ICI). Each VI schedule consisted of 12 intervals, drawn from the distribution reported by Catania and Reynolds (1968). These intervals were randomized before each experimental session. If the interreinforcement interval (IRI) did not end with a reinforcer at the end of a component, that same IRI was resumed at that same point during the next presentation of that component. To mitigate potential time‐based control effects, component durations of 2 or 4 min were employed (cf. Leslie, 1996, p. 201). This decision was informed by similar practices observed in Freeman & Lattal (1992, Experiment 3), where components alternated with durations of 15, 30, or 45 s. Components were separated by a 10‐s ICI, when the entire screen turned black and “WAIT!” (written in Portuguese) was presented in red in the center screen. In the lower reinforcement magnitude component (hereafter, the lean component), the monetary value delivered was R$0.02, and in the greater reinforcement magnitude component (hereafter, the rich component) it was $0.20. Participants were divided evenly and randomly assigned to start the training phase with the lean or rich component.

#### Phase 1: Baseline

During baseline (BL1), the conditions were identical to those during training, except that participants were exposed to 24‐min sessions under a multiple VI 30‐s VI 30‐s schedule of reinforcement (excluding the ICI). Each component was in effect for 3 or 6 min, separated by a 10‐s ICI, and the total time for each component was 12 min. The monetary value in the lean component was $0.05 and in the rich component $0.50. The BL1 lasted a maximum of 10 sessions or until differences in mean response rates of the first two and next two sessions, divided by the mean of these four sessions, were ≤ 15% in both components (cf. Costa & Cançado, 2012; Cumming & Schoenfeld, 1960).

#### Phase 2: Test 1

During the test (T1), the conditions were identical to those during BL1, except that the spring‐loaded button replaced the mouse as the operandum in both multiple schedule components. As noted in the apparatus section, the spring‐loaded button resulted in an increase in the response force requirement from approximately 1 N to 70 N.

The instructions (in Portuguese) were as in BL sessions, with only the *italicized* portion in the preceding instructions changed as follows:

The session will start when you press the button for the first time. By pressing the button in front of you, a smiley face will eventually appear in the upper right corner of the screen. When that happens, click on the ESC button, and a monetary value will be credited in the window at the screen's top center, above a colored button. The monetary value will not be credited if you do not press ESC.

#### Phase 3: Return to baseline

Return to baseline (BL2) was a replication of Phase 1.

#### Phase 4: Return to test

Return to test (T2) was a replication of Phase 2.

### Results

**FIGURE 1:**
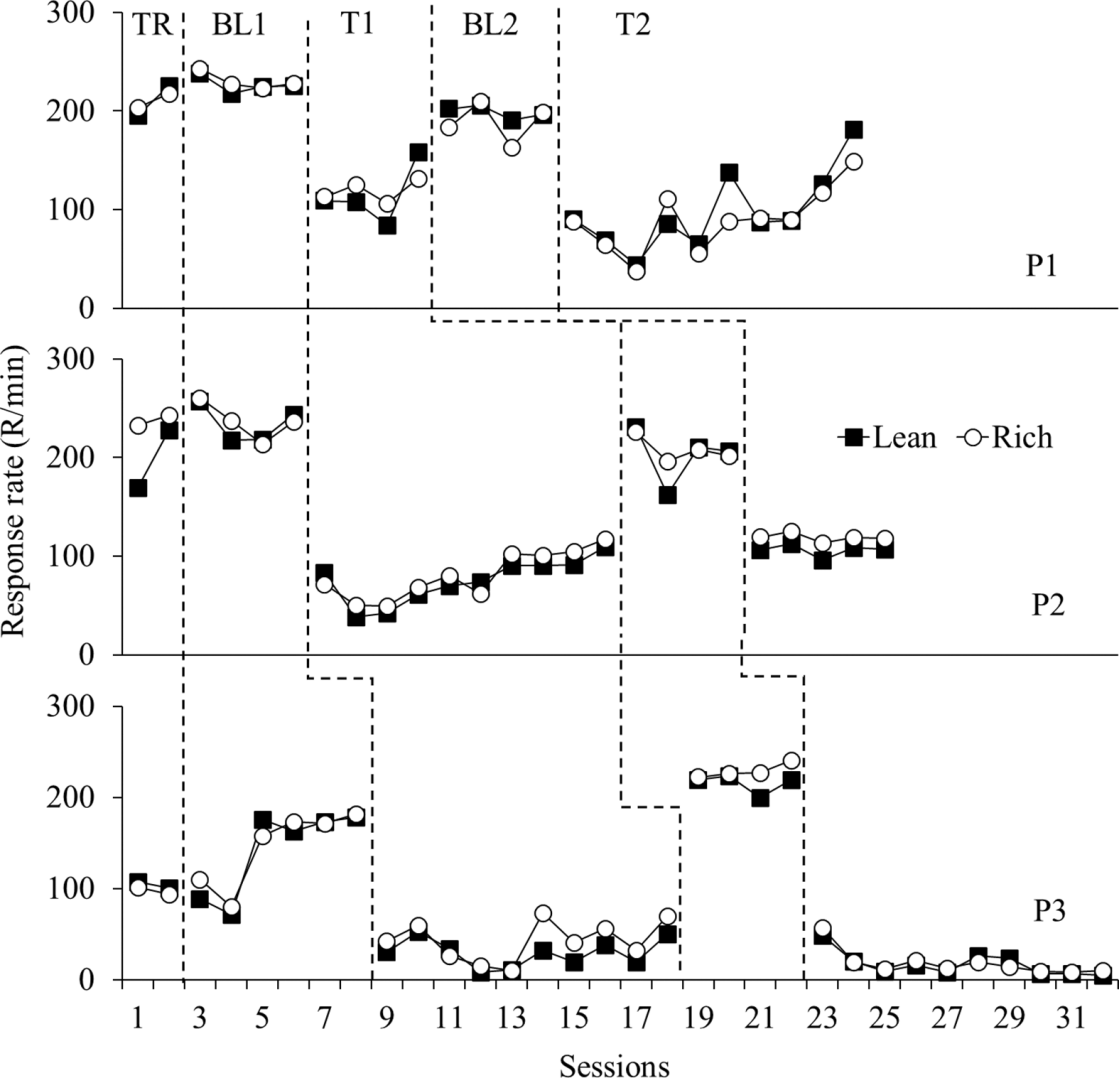
Response rates per minute (R/min) for each participant in each phase.

Figure 1 shows response rates (responses per minute, R/min) for each participant in each phase. Reinforcement rates were similar between the components for each participant (see data for the reinforcement ratio obtained between the components in Table 1). As can be seen in Figure 1, response rates were similar between the components of the multiple schedule for all participants during BL. During T1, with the addition of the spring‐loaded button, response rates decreased relative to BL in both components similarly for all participants.

**FIGURE 2:**
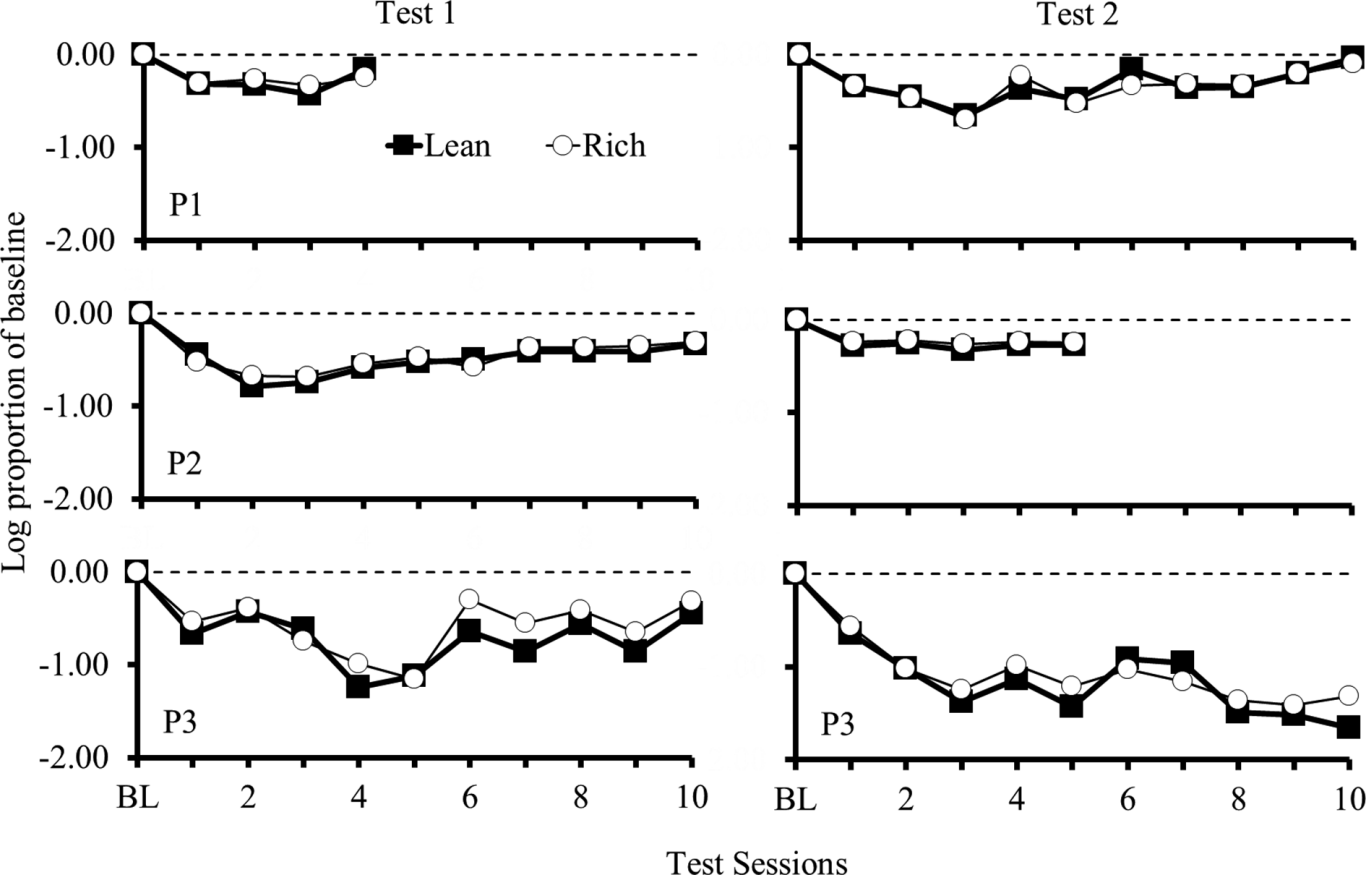
Log proportion of the mean response rate during the last four BL sessions across test sessions for all participants. The horizontal line at 0 indicates mean BL response rates. Greater deviations from 0 indicate greater changes in responding during the test relative to BL, suggesting less resistance to change.

Figure 2 shows log proportion of the mean of the response rate during the last four BL sessions across test sessions for all participants. Greater deviations from 0 indicate greater changes in responding during the test relative to BL, suggesting lower resistance to change. The data from Figure 2 do not indicate systematic differences in the resistance to change between responding during the components, except for the final five sessions of T1 for P3 in which resistance to change was greater during the rich component.

### Discussion

The different reinforcement magnitudes used in this experiment generated neither differential responding nor differential resistance to change. Among the possible reasons for these nondifferential effects are the characteristics of the reinforcer and the type of disruptor—increases in response force—used in this experiment. The latter is an unlikely cause of the outcome because during both test conditions (i.e., Phases 2 and 4), response rates decreased in both components for each participant relative to BL, corroborating the results of nonhuman animal (e.g., Alling & Poling, 1995; Chung, 1965) and human experiments (Costa et al., 2024). Thus, increasing physical effort is a viable disruptive operation in research on resistance to change in humans.

The operandum changed from a mouse to the force operandum described above between the baseline and the resistance test. This raises the question of the possible role of this change in the results. Costa et al. (2024, Experiments 2 and 3) addressed this issue by showing that the results of resistance to change tests involving different reinforcement rates were equivalent regardless of whether the operandum was the same or different in the baseline and resistance test conditions.

Two characteristics of the reinforcer that may have contributed to the negative results of this experiment are the ratios of the reinforcer magnitude differences between the two components (1:10 in this experiment) and the accessibility of the reinforcer for consumption. The magnitude of reinforcement may be a more salient stimulus when the manipulated values of reinforcer magnitude are large (e.g., Bonem & Crossman, 1988; cf. Lerman et al., 2002). Therefore, a change to be tested would be that of increasing the ratios of the reinforcer magnitude differences between the two components (e.g., 1:20).

In nonhuman animal experiments, subjects can often consume the reinforcers, such as food or water, immediately in the operant chamber sessions and have direct access to the reinforcer of the target response. In Experiment 1, responses increased a counter by different values depending on the component. Because the counter value was exchanged for real money only at the end of the session, these reinforcers could not be immediately consumed following a response in the experimental space. This situation is different from that with experiments with nonhuman animals, where reinforced responses result in immediate access to a primary reinforcer. Although points exchangeable for money have been used as reinforcers for human responding in several experiments (e.g., Costa et al., 2013; Cunha et al., 2018; Luiz et al., 2020, 2021; Porto et al., 2011; Weiner, 1963), there is no evidence that such points affect human resistance to change as a function of reinforcement magnitude differences. Given these considerations, Experiment 2 was designed to investigate resistance to change as function of the magnitude of reinforcement using a larger magnitude difference between the reinforcers in either component of the multiple schedules and with reinforcers that were consumable when earned as opposed to the delayed contact required by the procedure of Experiment 1.

## EXPERIMENT 2

This experiment was similar to Experiment 1, except that differing amounts of snacks or money were delivered as reinforcers immediately on completion of the required response in either component of a multiple VI VI schedule.

### Method

#### Participants and apparatus

Four undergraduate students (P4–P7), one male and three females, 18–23 years old, participated in Experiment 2. The invitation, locale, and apparatus were as described in Experiment 1.

#### Procedure

The procedure was the same as that for Experiment 1, with a few exceptions: (a) an experimenter located inside the experimental room delivered coins (to P4 and P5) or snacks (to P6 and P7) to the participants (assignment of participants to the two procedures was random), (b) the reinforcers were presented in plastic cups (for money) and on napkins (for snacks), (c) there was no point counter on the computer screen, (d) the proportional difference in monetary reinforcement magnitude was increased from 1:10 (Experiment 1) to 1:20, and (e) the duration of the sessions was 12 min.

Points (a) and (b) above were modified because no apparatus could automatically deliver the reinforcements and the experimental room did not allow for an arrangement in which the experimenter could stay in an adjacent room to deliver the reinforcements through a conveniently constructed opening. Removing the point counter ensured participants' direct contact with the monetary reinforcer or snacks without mediation. The duration of the sessions was reduced to ensure that there would be relatively few reinforcements in the snack procedure to preclude satiation (each participant received between 40 and 44 snacks per session). To ensure that the snacks could function as reinforcers, the following procedures were adopted as establishing operations: (a) sessions were scheduled during lunchtime, and participants were instructed not to eat for at least 3 hr. prior to the session and (b) participants selected three of 14 snacks of their choice to be consumed (more details below).

Table 2 shows the phases of Experiment 2 for each participant, the multiple schedule used, the reinforcer type and value for each multiple schedule component, the reinforcement ratio obtained between the components, and the operandum used. The number of sessions in each phase is shown in parentheses.

**TABLE 2:** Procedure for Experiment 2.

	Coins		Snacks	
Phase	P4	P5	P6	P7
TR	VI 10 s VI 10 s	VI 10 s VI 10 s	VI 10 s VI 10 s	VI 10 s VI 10 s
	R$0.01 / R$0.25	R 0.01 / R$0.25	1 snack/ 2 snacks	1 snack/ 2 snacks
	1.00	1.00	1.00	1.00
	Mouse 1 N	Mouse 1 N	Mouse 1 N	Mouse 1 N
	(1)	(1)	(1)	(1)
	VI 20 s VI 20 s	VI 20 s VI 20 s	VI 20 s VI 20 s	VI 20 s VI 20 s
	R$0.01 / R$0.25	R$0.01 / R$0.25	1 snack/ 2 snacks	1 snack/ 2 snacks
	1.00	1.00	1.00	1.00
	Mouse 1 N	Mouse 1 N	Mouse 1 N	Mouse 1 N
	(1)	(1)	(1)	(1)
BL	VI 30 s VI 30 s	VI 30 s VI 30 s	VI 30 s VI 30 s	VI 30 s VI 30 s
	R$0.05 / R$1.00	R$0.05 / R$1.00	1 snack/ 3 snacks	1 snack/ 3 snacks
	1.00	1.00	1.00	1.00
	Mouse 1 N	Mouse 1 N	Mouse 1 N	Mouse 1 N
	(4)	(10)	(4)	(7)
T	VI 30 s VI 30 s	VI 30 s VI 30 s	VI 30 s VI 30 s	VI 30 s VI 30 s
	R$0.05 / R$1.00	R$0.05 / R$1.00	1 snack/ 3 snacks	1 snack/ 3 snacks
	1.00	1.00	1.00	1.00
	Spring 70 N	Spring 70 N	Spring 70 N	Spring 70 N
	(4)	(3)	(4)	(4)

*Note*: TR = training; BL = baseline; T = test; N = Newtons. The monetary value shown on the screen was in Brazilian currency (R$). At the time the experiment was conducted, R$0.05 was approximately equivalent to US$0.015. The number below the monetary values indicates the reinforcement ratio obtained between components of the multiple schedule (more details in the text).

#### Monetary value procedure

Participants P4 and P5 were exposed to this procedure. During the training phase (described below), the reinforcer in the lean component was $0.01 and in the rich $0.25. During the BL phase (described below), the reinforcer for the lean was $0.05 and for the rich $1.00. When a response met the schedule requirement, a smiley face appeared below the consummatory‐response button, signaling the availability of the reinforcer. The smiley face remained until the participant clicked the consummatory response button (training and BL phases) or pressed the Escape key on the keyboard (test phase). Immediately thereafter, the experimenter deposited a coin corresponding to the magnitude of reinforcement of the schedule inside a red or green plastic cup, depending on the component of the multiple schedule in effect. The time between the appearance of the smiley face and pressing the button—the consummatory response button or Escape key—was not recorded for the session (i.e., reinforcement delivery paused the timers).

#### Snacks procedure

Participants P6 and P7 were exposed to this procedure. During the training phase (described below), the reinforcer was one snack in the lean component and two snacks in the rich component. During the BL phase (described below), the reinforcer was one snack in the lean component and three snacks in the rich component. Before each session, 14 snack options (e.g., peanuts, Brazil nuts, pieces of chocolate, popcorn, pieces of marshmallow, raisins etc.) were presented to the participants, who chose two or three options that they wished to receive during the experimental sessions. When a response met the schedule requirement, the smiley face described above appeared below the consummatory response button, signaling the availability of the reinforcer. Immediately after that, the experimenter deposited the corresponding number of snacks for the different schedules on a green or red napkin depending on the multiple schedule component in effect. The participants had to consume (i.e., eat) the earned snacks and then click on the consummatory response button (training phase and BL) or press the Escape key on the keyboard (test phase) to make the smiley face disappear. The time between the appearance of the smiley face and pressing the button—either the consummatory response button or the Escape key—was not counted for the session (i.e., reinforcement delivery paused the timers). Water was available to the participants during the ICI.

#### Training phase

Before the two training sessions, all the participants were asked to read the following instructions (in Portuguese).

This study is not about intelligence or personality. To start the session, click the mouse cursor on the button that will appear at the bottom center of the screen [Start Session]. By clicking with the mouse cursor on the response button (green or red central button), eventually, a smiley face will appear in the upper right corner of the screen.

After the initial instructions above, for P4 and P5, the instructions were as follows:

When that happens, click on the button in the monitor's upper right corner (above the smiley face), and the experimenter will hand you a coin. The experimenter is not authorized to give any additional information. Good work!

After the initial instructions above, for P6 and P7, the instructions were as follows:

When that happens, the experimenter will give you something to eat (snacks). Immediately after the immediate consumption of the snack, click on the button in the upper right corner of the monitor (above the smile). The experimenter is not authorized to give any additional information. Good work!

Initially, the participants were exposed to one session of a multiple VI 10‐s VI 10‐s schedule followed by a second session in which a multiple VI 20‐s VI 20‐s schedule was in effect. The training phase was identical to that for Experiment 1, except for the reinforcers used and the ICI, which in this experiment was 1 min. The components occurred twice in each session in simple alternation (e.g., lean‐rich‐lean‐rich or in reverse order).

#### BL phase

The conditions were as in the training phase, except participants were exposed to a multiple VI 30‐s VI 30‐s schedule with each schedule consisting of the same intervals used in Experiment 1. Components lasted for 2 or 4 min, and each session lasted for 12 min (excluding the ICI and reinforcement time, described below). The total time for each component across each session was the same, 6 min.

#### Test phase

The test phase was the same as the BL, except the spring‐loaded button was added as the operandum to all components simultaneously, increasing the response force required to respond, as noted in Experiment 1. Before the test sessions, participants were asked to read the following instructions (in Portuguese).

The session will start when you press the button for the first time. By pressing the button in front of you, a smiley face will eventually appear in the upper right corner of the screen.

After the initial instructions above, for P4 and P5, the instructions were as follows:

When that happens, click on the ESC button, and the experimenter will hand you a coin. The experimenter is not authorized to give any additional information. Good work!

After the initial instructions above, for P6 and P7, the instructions were as follows:

When that happens, the experimenter will give you something to eat (snacks). Immediately after the immediate consumption of the snack, click on the ESC button. The experimenter is not authorized to give any additional information. Good work!

### Results

**FIGURE 3:**
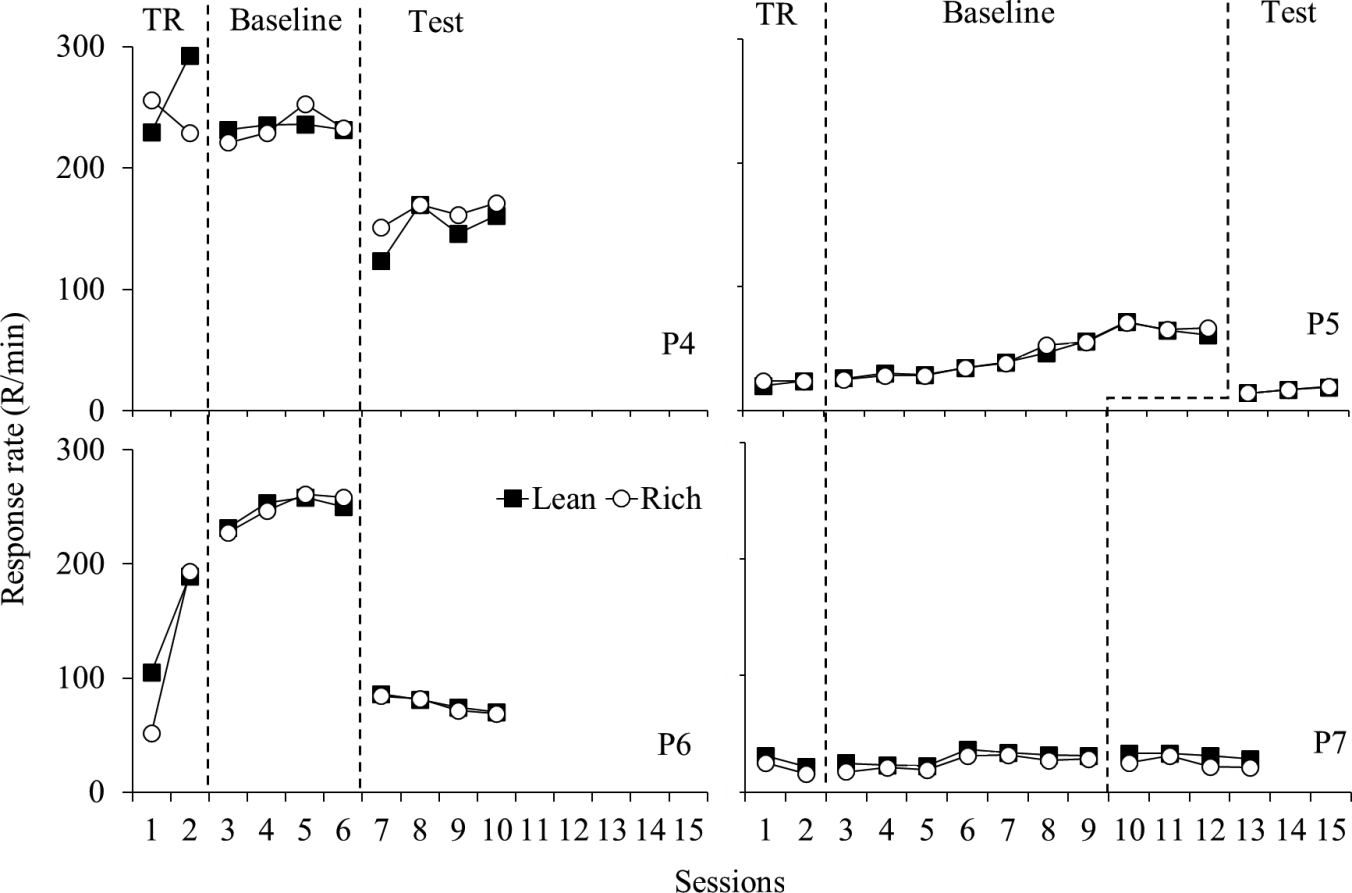
Response rates (R/min) for each participant in the three phases of Experiment 2. Participants P4 and P5 were exposed to the monetary‐value procedure, and P6 and P7 were exposed to the snacks procedure.

Figure 3 shows response rates (R/min) for each participant in the three phases of Experiment 2. Reinforcement rates were similar between the components for each participant (see data for the reinforcement ratio obtained between the components in Table 2). During BL, response rates were similar between the components for each participant. During test sessions, response rates decreased relative to BL for each participant except P7. Substituting the spring‐loaded button for the mouse similarly affected responding in both components.

**FIGURE 4:**
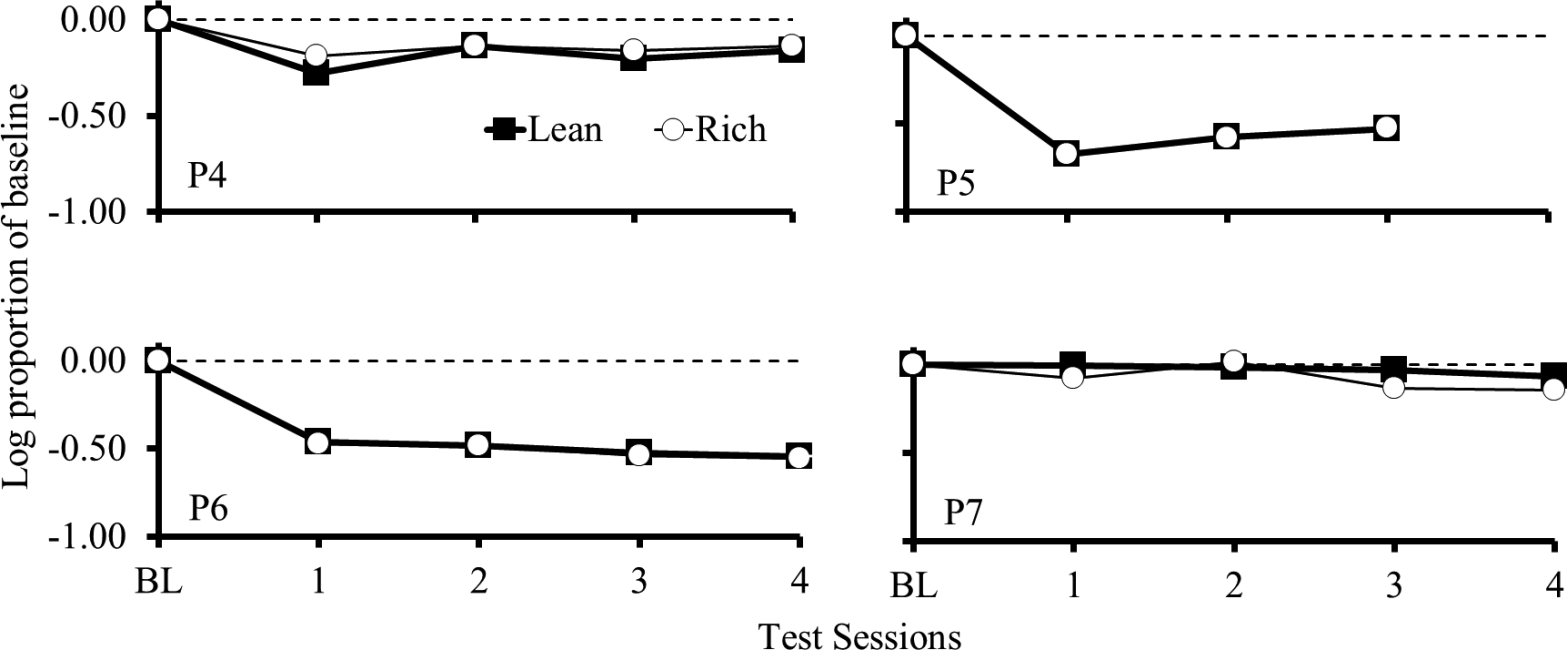
Responding during each test session as a log proportion of mean response rate during the last four BL sessions for each participant in Experiment 2. Participants P4 and P5 were exposed to the monetary‐value procedure, and P6 and P7 were exposed to the snacks procedure.

Figure 4 shows responding during each test session as a log proportion of the mean of the response rate during the last four BL sessions for each participant. Greater deviations from 0 indicate greater changes in responding during the test relative to BL, suggesting a lower resistance to change. As in Experiment 1, the data in Figure 4 do not indicate systematic differences in resistance to change between the rich and lean components.

### Discussion

As in the first experiment, there were no systematic differences in resistance to change as a function of differences in the magnitude of reinforcement when the ratio of the difference between the larger and smaller reinforcer was increased or when the reinforcers were either immediately available snacks or coins instead of points exchangeable later for money. As in the first experiment, these results do not corroborate the general findings of related experiments with nonhuman animals (e.g., Dulaney & Bell, 2008; Harper, 1996; Harper & McLean, 1992, Experiment 1; Nevin, 1974, Experiment 3) reporting differences in resistance to change of responding maintained by different reinforcement magnitudes.

Sidman (1960) observed that the generality of any result is a product of the precision of the experimental procedures in effect. In the present case, for example, a failure to replicate resistance to change effects as a function of reinforcement rate using the same participants and the same disruptor would suggest a problem with the present procedures, whereas a replication of resistance to change effects with different reinforcement rates would suggest that something other than the procedures per se was responsible for the differences.

Therefore, Experiment 3 was conducted as a systematic replication of Experiment 2 but with a variable previously shown to affect resistance to change of responding by humans—differential rates of reinforcement (Costa et al., 2024). This manipulation thus provided a test for potential weaknesses in the procedures used in the first two experiments that might have precluded finding a systematic relation between reinforcement magnitude and resistance to change.

## EXPERIMENT 3

The purpose of Experiment 3 was to examine the effects of reinforcement rate on resistance to change of humans engaged in a computer task for money, snacks, and points exchangeable for money.

### Method

#### Participants and apparatus

The participants and apparatus were the same as in Experiment 2.

#### Procedure

Table 3 summarizes the conditions of Experiment 3 for each participant, the multiple schedules used, the mean (standard deviations in parenthesis) of received reinforcers for each component of multiple schedules, the reinforcer type and value for each component of multiple schedules, the operandum used, and the number of sessions in each phase (in parentheses).

**TABLE 3:** Procedure for Experiment 3.

		Coins		Snacks	
	Phase	P4	P5	P6	P7
Condition 1	BL1	VI 10 s VI 100 s	VI 10 s VI 100 s	VI 10 s VI 100 s	VI 10 s VI 100 s
		88.0 (*0.8)* 9.0 (*0.0*)	85.6 (*1.7)* 9.2 (*0.8*)	88.5 (*0.6)* 8.8 (*0.3*)	82.2 (*0.8)* 8.8 (*0.4*)
		R$0.10	R$0.10	1 snack	1 snack
		Mouse 1 N	Mouse 1 N	Mouse 1 N	Mouse 1 N
		(4)	(5)	(4)	(5)
	T1	VI 10 s VI 100 s	VI 10 s VI 100 s	VI 10 s VI 100 s	VI 10 s VI 100 s
		86.8 (*0.5)* 8.5 (*1.0*)	79.3 (*0.5)* 9.0 (*0.8*)	86.3 (*1.0)* 8.8 (*0.5*)	80.8 (*1.5)* 8.5 (*0.6*)
		R$0.10	R$0.10	1 snack	1 snack
		Spring 70 N	Spring 70 N	Spring 70 N	Spring 70 N
		(4)	(4)	(4)	(4)
Condition 2	BL2	VI 10 s VI 100 s	VI 10 s VI 100 s	VI 10 s VI 100 s	VI 10 s VI 100 s
		84.5 (*3.3)* 8.5 (*0.6*)	86.3 (*1.5)* 8.8 (*1.0*)	87.8 (*1.0)* 8.8 (*0.5*)	85.3 (*0.5)* 9.0 (*0.0*)
		100 points	100 points	100 points	100 points
		Mouse 1 N	Mouse 1 N	Mouse 1 N	Mouse 1 N
		(4)	(4)	(4)	(4)
	T2	VI 10 s VI 100 s	VI 10 s VI 100 s	VI 10 s VI 100 s	VI 10 s VI 100 s
		81.3 (*4.9)* 8.3 (*0.5*)	79.0 (*1.4)* 8.3 (*0.5*)	86.0 (*1.4)* 8.5 (*0.6*)	82.8 (*2.0)* 8.5 (*0.6*)
		100 points	100 points	100 points	100 points
		Spring 70 N	Spring 70 N	Spring 70 N	Spring 70 N
		(4)	(4)	(4)	(4)

*Note*: TR = training; BL = baseline; T = test; N = Newtons. The monetary value shown on the screen was in Brazilian currency (R$). At the time the experiment was conducted, R$0.05 was approximately equivalent to US$0.015. The means (standard deviations in parentheses) of received reinforcers for each component of multiple schedules are exhibited above the monetary value.

Experiment 3 consisted of the following conditions in which each participant was exposed to BL and test for Condition 1, then BL and test for Condition 2.

#### Condition 1: Snacks and monetary values

The invitation to the participants, experimental setting, computer‐screen layout, and apparatus were as in the BL condition of Experiment 2, except all participants were exposed to 30‐min sessions under a multiple VI 10‐s VI 100‐s schedule of reinforcement (rich and lean components, respectively). The IRIs were randomized in both components so that the longest IRI of the rich component schedule (19 s) was smaller than the smallest IRI of the lean component schedule (68 s) (cf. Lacerda et al., 2017, p. 145; Mace et al., 1990, p. 166). The VI 10‐s schedule consisted of the following intervals: 2, 5, 6, 8, 11, 13, 16, and 19 s. The VI 100‐s schedule consisted of the following intervals: 68, 72, 80, 88, 98, 104, 110, and 180 s. The reinforcer magnitude remained constant throughout the experiment. Participants P4 and P5 received a $0.10 coin in both components, and P6 and P7 received one snack in both components. Each component was in effect for 5‐min periods, separated by a 60‐s ICI, with the first component selected randomly. Test 1 (T1) was the same as the BL, except the spring‐loaded button was added to both components simultaneously. This increased the response force requirement to respond to 70 N. The mouse was removed. The test phase consisted of four sessions. The experimental task during T1 was the same as in the test described in Experiment 2: When a response met the schedule requirement, P4 and P5 received $0.10 in both components and P6 and P7 received one snack.

#### Condition 2: Points exchangeable for money

In the next session, Condition 2 was initiated after T1 in Condition 1. The BL2 was the same as in Condition 1, except a 10‐s ICI separated the components and there was a point counter above the response button on the computer screen (details below). The participants were asked to read the following instructions (in Portuguese). The part of the text in *italics* changed before initiating the test sessions. However, there were no *italics* when presented to the participants.

This study is not about intelligence or personality. *To start the session*, *click the mouse cursor on the button that will appear at the bottom center of the screen [Start Session]. By clicking the mouse cursor on the response button (green or red central button)*, *a smiley face will eventually appear in the upper right corner of the screen. When this happens*, *click on the button in the upper right corner of the monitor (above the smiley face)*, *and 100 points will be added in the window in the screen's upper center*, *above the response button (green or red)*. Every 100 points obtained will be exchanged for R$0.10 at the end of each session. The experimenter is not authorized to give any additional information. Good work!

Test 2 (T2) was the same as BL2, except the spring‐loaded button replaced the mouse as the operandum. The T2 phase consisted of four sessions. Instructions were like those in BL2 sessions, with only the italicized portion (described above) replaced by the instructions below (in Portuguese):

The session will start when you press the button for the first time. By pressing the button in front of you, a smiley face will eventually appear in the upper right corner of the screen. When that happens, click on the ESC button, and 100 points will be credited in the window at the screen's top center, above the response button (green or red).

In the BL2 and T2 phases, when a response met the schedule requirement, an image of a smiley face appeared below the consummatory response button, signaling the availability of one reinforcer (100 points). The smiley face remained until the participant clicked the consummatory response button (BL2 phase) or pressed the Escape key on the keyboard (T2 phase) to add the 100 points to the counter. At the end of each session, the screen showed the total points gained and the message “Call the Experimenter.” The participants were paid for their performance at the end of each session.

### Results

**FIGURE 5:**
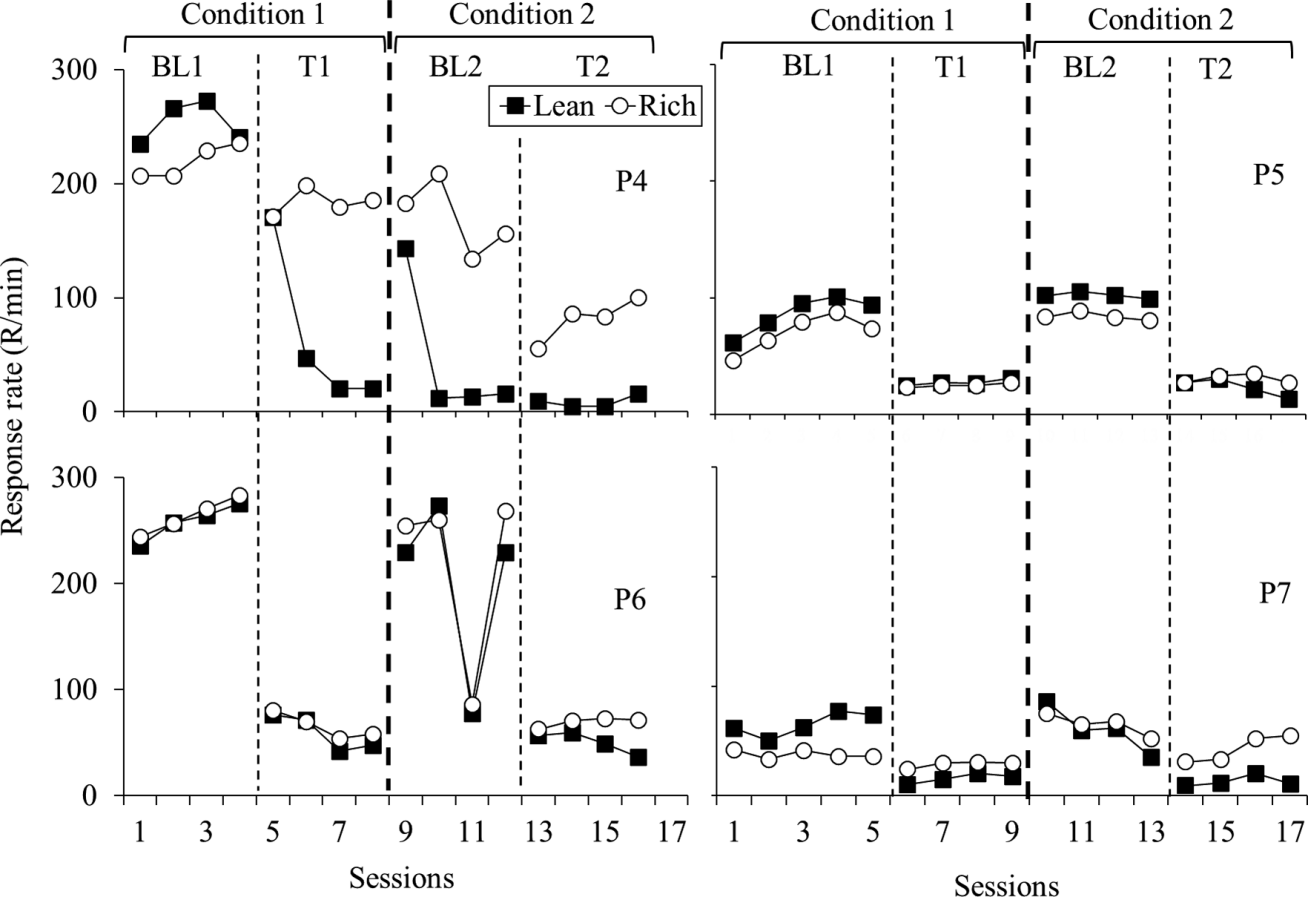
Response rates (R/min) for each participant during Experiment 3. Participants P4 and P5 were exposed to the monetary‐value procedure, and P6 and P7 were exposed to the snack procedure in Condition 1. All participants were exposed to points that were exchangeable for money in Condition 2.

Figure 5 shows response rates (R/min) for each participant during Experiment 3. Reinforcement rates were higher in the rich component during BL1 (see the data for mean received reinforcers between the components of multiple schedules in Table 3). Response rates were higher during BL1 (Condition 1) in the lean component for all participants except P6. During T1, response rates decreased relative to the BL1 for each participant in both VI components. During BL2 (Condition 2), response rates were similar between the components for P6 and P7 and greater in the VI 10 s for P4 and the VI 100 s for P5. During T2, response rates decreased in both components for each participant.

**FIGURE 6:**
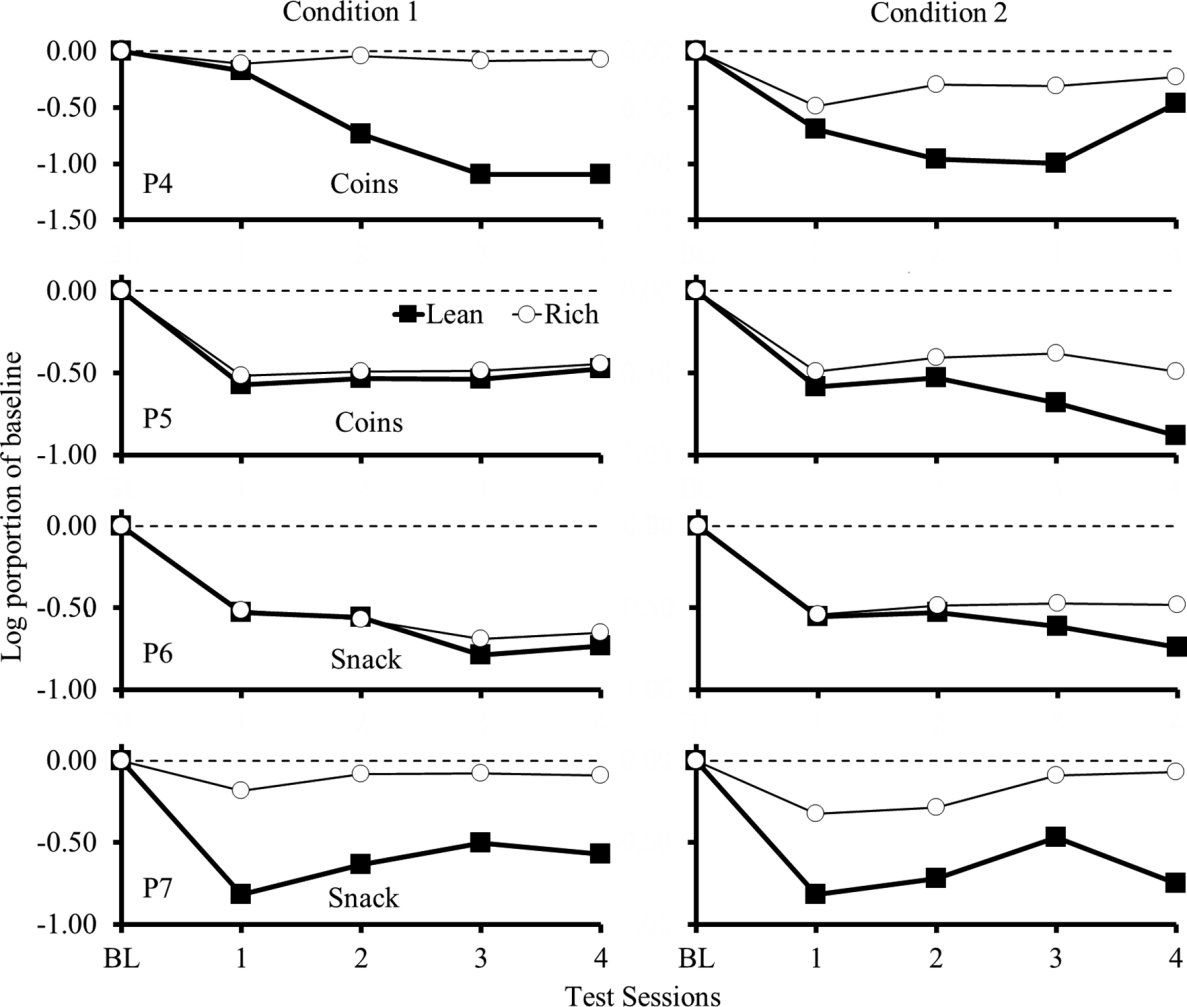
Log proportion of responding during each test session as the log proportion of the mean response rate during the last four BL sessions for each participant in Experiment 3. Participants P4 and P5 were exposed to the monetary‐value procedure, and P6 and P7 were exposed to the snack procedure in Condition 1. All participants were exposed to points that were exchangeable for money in Condition 2.

Figure 6 shows the log proportion of responding during each test session as the log proportion of the mean response rate during the last four BL sessions for each participant. Greater deviations from 0 indicate greater changes in responding during the test relative to BL, suggesting a lower resistance to change. The *y*‐axis differs between P4 and the other participants.

In Condition 1, P4 and P7 exhibited greater persistence of responding in the rich component. Participant P5 showed slightly greater persistence in the rich component, and P6 also showed greater persistence in the rich component, but only during the last two sessions of T1. In Condition 2, each participant showed greater persistence in the rich component, except during the first test session for P6.

### Discussion

In general, there was greater resistance to change in the rich than in the lean component for each participant, but not in every instance. These findings corroborate previous findings using both nonhuman and human animals in which resistance to change was found to be a function of reinforcement rate (e.g., Cohen, 1996; Costa et al., 2024; Igaki & Sakagami, 2004; Mace et al., 1990; Nevin, 1974, Experiments 1 and 2). These results indicate that the absence of systematic changes in responding in the rich and lean components as a function of different reinforcement magnitudes was not the result of experimental control issues. The same participants served in both Experiments 2 and 3, further supporting the latter observation. Although the resistance of these participants' responding was insensitive to differences in reinforcement magnitude in Experiment 2, the same participants' responding, under otherwise very similar conditions in Experiment 3 was differentially resistant to the disruptive effects of increased force when it was maintained by different rates of reinforcement.

Furthermore, the results shown in Figure 1 (Experiment 1), Figure 3 (Experiment 2), and Figure 5 (Experiment 3) collectively establish that transitioning from the computer mouse to the loaded‐spring button, which necessitates greater physical effort to register a response, similarly decreased responses maintained by VI schedules arranging either different magnitudes or rates of reinforcement.

## GENERAL DISCUSSION

As in previous experiments, resistance to change of responding of human subjects in Experiment 3 varied as a function of reinforcement rate of such responding. However, in both Experiments 1 and 2, neither responding nor the resistance to change of those rates was differentially affected by different magnitudes of reinforcement. This was the case despite that (a) the participants were the same in Experiments 2 and 3 and (b) different reinforcement magnitudes were arranged in different ways (Experiments 1 and 2). The results of Experiments 1 and 2 raise issues about the nature and generality of reinforcement magnitude effects on response rate and those effects on resistance of human operant responding to change. Each of these are discussed below, followed by a consideration of some methodological issues common to all three experiments.

### Definitions of reinforcer magnitude

As noted in the introduction, reinforcement magnitude is a generic variable that subsumes several different procedures, making it somewhat challenging to draw general conclusions about the unitary effects of reinforcement magnitude on either response maintenance or resistance to change (see, e.g., Bonem & Crossman, 1988, for a review of the various procedures subsumed under the label of “reinforcement magnitude” and the disparities in reinforcement magnitude effects). Much of the literature on resistance to change involves research with pigeons, where the reinforcer magnitude is defined as different durations of access to a food hopper. Comparing these magnitude effects to those obtained with humans invites consideration of motivating operations and economic variables.

Unlike experiments investigating reinforcement magnitude with nonhumans, with humans there is less control over motivating/establishing operations (cf. Laraway et al., 2003; Michael, 1993) and economic variables (cf. Hall & Lattal, 1990; Hursh, 1984). With the analysis of human behavior, the use of points as reinforcers can be enhanced by using procedures like those in Experiment 1, where points could be exchanged for money after the procedure was completed. The immediate availability of the reinforcer was enhanced in Experiment 2, where snacks and money were available immediately, albeit intermittently, following target responses. A further attempt to control the establishing operation in Experiment 2 was undertaken by giving participants receiving snacks choices between them and by arranging sessions when hunger was likely (lunch time). The success of such attempts, however, was not assessed and it is possible that the failure to differentially establish responding with the different magnitudes was in part due to the lack of control over the motivating/establishing operations. That the establishing operation was not responsible for the failure to obtain differential resistance to change in Experiments 1 and 2 is demonstrated in that the same establishing operations produced differential resistance to change when measured against different reinforcement rates in Experiment 3 as opposed to the different reinforcement magnitudes in Experiments 1 and 2.

The economic context in which the reinforcers occurred in the present experiments and that in effect during the typical behavioral momentum theory experiment involving reinforcement magnitude with nonhumans differ, which also may have contributed to the different outcomes. In a closed economy, access to a reinforcer is restricted to the experimental situation, but in an open one, the same reinforcer is available both in the experimental situation and outside it (Hall & Lattal, 1990; Hursh, 1984). In practice the “closedness” or “openness” of most economies in research settings are relative and fall on a continuum between the two (Imam, 1993). Experimental animals are sometimes fed after a session to maintain their body weights, thereby opening the economy relative to no such postsession feeding. Also, open economies can be more or less restrictive with respect to within‐session reinforcer availability outside of sessions. In experimental situations involving reinforcement, humans receive their within‐session reinforcers in a more open economic context than do food‐restricted experimental animals (but see Roane et al., 2005). In the present experiments, humans' access to money and food were not restricted, providing another potential difference between the present results and earlier experiments involving reinforcement magnitude and behavioral momentum theory.

### Reinforcer magnitude and response rates

Even within a single definition of reinforcement magnitude, there are failures to differentially maintain responding with formally defined different reinforcement magnitudes. Based on their analysis of variations of reinforcement duration (access to grain by pigeons) in different components of a multiple schedule, Shettleworth and Nevin (1965) similarly concluded that there was “no entirely consistent relation between absolute rate of responding and absolute magnitude of reinforcement” (p. 200). Also, Nevin (1974, p. 396) found “no consistent differences in response rates with 7.5‐sec and 2.5‐sec reinforcement durations” with one of two pigeons in his Experiment 3. Harper and McLean (1992, Experiment 1) found higher response maintained by a 6‐ than 2‐s duration reinforcer delivered according to a VI 2‐min schedule in 75% of their comparisons. The reasons for these failures are beyond the scope of the present discussion in that they may relate to the ratios of amount of food consumed in relation to the reinforcer duration access. The relevant point for the present experiments is that these findings with humans and a different form of reinforcement magnitude are not inconsistent with at least some of the extant research on magnitude of reinforcement effects.

How reinforcement magnitudes are arranged can affect the outcomes of these variables. Catania (1963, see also Neuringer, 1967) showed that the same differences in two reinforcement magnitudes differentially affected responding when arranged in different components of a concurrent schedule but not when arranged across successive conditions. The present experiment required using multiple schedules to make the results comparable to those of previous investigations of resistance to change as a function of both magnitude (Nevin, 1974, Experiment 3) and other reinforcement parameters (Nevin, 1974, Experiments 1 and 2).

### Reinforcer magnitude and resistance to change

In the research cited throughout this article, the relation between reinforcement magnitude and resistance to change has proven to be complicated. In some cases, there is a clear difference in resistance to change as a function of the duration of the reinforcer (e.g., Harper & McLean, 1992; Nevin, 1974, Experiment 3). In others (e.g., McComas et al., 2008), the effect of reinforcement magnitude seems to depend on the context in which it is studied. Sometimes, too, differential resistance to change has been found even when the different magnitudes do not differentially maintain responding. Nevin (1974, Experiment 3) found differences in resistance to change as a function of long and short reinforcer‐access durations in a pigeon with nondifferentiated responding maintained by the different reinforcer‐access durations, as did Harper and McLean (1992) in some of their subjects (Pigeon C7 with 120 dark‐key reinforcers per hour). In the present experiment, both nondifferential responding and nondifferential resistance to change occurred in both Experiments 1 and 2.

### Methodological issues

The present experiments raise several methodological issues. The first is the absence of orderly functional relations between reinforcer magnitude and response rate in the present participants. As noted, this relation is complicated and not always systematic (Bonem & Crossman, 1988). A relevant question, but one beyond the scope of these experiments, is whether the different magnitudes of reinforcement investigated here would yield differential responding when scheduled differently, perhaps as components of a concurrent schedule, as in Catania (1963). Although it would be useful to know more about the reinforcement magnitude effects under those conditions, whether such effects would inform the understanding of human performance under the different reinforcement magnitudes in the multiple schedules investigated in the present experiments is similarly unknown.

Other methodological questions related to all three of the present experiments involve the relation between the button press and the spring‐loaded operandum. One is whether the change from the button press during the training conditions to the spring‐loaded operandum during the testing conditions affected the outcomes. First, despite that changes in operanda occurred in all three experiments, only reinforcement magnitude, not reinforcement rate, failed to produce differential resistance to change, suggesting it is not the operandum but the reinforcement parameter that is responsible for the different results. Second, changing the operandum from a mouse to the spring‐loaded button might result in a shift of control by the computer monitor, which in turn might affect the monitoring of reinforcement accumulation. That this was not the case is suggested by the results of Experiment 3 in comparison to those of Experiments 1 and 2, as noted above. Furthermore Costa et al. (2024) found effects similar to those of Experiment 3 when there was either a change or no change in the operandum between the baseline and test conditions.

Another question related to the use of two operanda was whether participants pressed the spring‐loaded button and the escape key simultaneously in test conditions (which could not be done in baseline conditions when the mouse was used as the operandum). By pressing the escape key (i.e., the consummatory response button) simultaneously with the spring‐loaded button, the participant could not be strictly under the control of obtaining reinforcers. Therefore, such “double pressing” might bias the findings. Based on unsystematic observations and a limited data set from one participant in Experiment 1, there was no evidence that this occurred. With the one participant in Experiment 1 for whom systematic data were not lost, the participant pressed the escape key the same number of times that they received reinforcers, suggesting that this participant did not press the escape key frequently along with the response button. When smileys appeared, the participant pressed only the escape key. This temporal proximity of the appearance of the smiley and the occurrence of the consummatory response attests to the participants' attentive engagement with the screen and the occurrence of the reinforcer. In Experiment 2, the researcher's presence in the room with the participant ensured precise monitoring of the entire execution of the experimental task. The experimenter could directly observe that participants only pressed the escape key after (a) the smiley face appeared on the computer screen and (b) the experimenter handed the snack or coin to the participant. Neither simultaneous responding on both operanda nor frequent pressing of the escape key were observed. Furthermore, video recordings of the sessions were made at the time of data collection. The researchers watched a small sample of each session, and it was possible to see that the participants issued the consumption response only after the smile appeared on the monitor (i.e., they did not keep pressing the ESC key). Unfortunately, the videos were discarded after the experiment was completed. Based on these different observations, simultaneous responding on both operanda was minimal, if it ever occurred at all.

## CONCLUSIONS

Negative results, like those obtained in Experiments 1 and 2, always are difficult to interpret. It may be that reinforcer magnitude is simply a less reliable variable in producing differential resistance to change than are other parameters of reinforcement. Another possibility is that despite the seeming structural similarities of the present procedures to the multiple‐schedule arrangements of both Nevin (1974, Experiment 3) and Harper and McLean (1992), there may have been subtle methodological differences between the different experiments that were at play. Experiment 3 eliminated both participant variables and the type of disruptor as the reasons for those results. The results of Experiment 3, along with the earlier results of Costa et al. (2024), suggest that the negative results of Experiments 1 and 2 were not the result of changing from the mouse to the spring‐loaded button as the operandum during the resistance to change test. However, other issues remain. Although there is a substantial literature on resistance to change in humans, practical issues preclude creating the kinds of exposure to motivating operations that are qualitatively equivalent to those used to establish reinforcers in nonhumans when studying magnitude and resistance to change. Creating nominally equivalent manipulations of reinforcer magnitudes in humans and pigeons may be fraught with complexities that may also have contributed to the different outcomes of the present experiments and at least some of those with nonhumans.

The results of several experiments suggest that organisms are more sensitive to the frequency than to the magnitude of reinforcement (e.g., Landon et al., 2003; Paula, 2016; Schneider, 1973; Todorov, 1973; Todorov & Ferreira, 1975; Todorov et al., 1984; see review by Bonem & Crossman, 1988). For example, Todorov (1973) varied the frequency and magnitude of reinforcement earned by pigeons in a concurrent VI VI schedule, finding that rate of reinforcement had stronger effects on choice behavior than did magnitude. Paula (2016) obtained similar results using a multiple VI VI schedule. The combined results of the present set of three experiments and those that have examined the sensitivity to the rate or magnitude of reinforcement suggest that response rate, choice, and resistance to change are generally more strongly affected by the rate than the magnitude of reinforcement.

Finally, although the results were negative with respect to the nominal differences in reinforcer magnitudes, the two magnitudes (a) controlled response rates similarly and (b) resulted in similar disruptions when response force was increased. Such results are consistent with those of previous investigations of resistance to change: If the maintaining conditions are functionally identical, as they were in the present Experiments 1 and 2, then imposing similar disruptors would be expected to produce similar effects, which is what occurred.

## AUTHOR CONTRIBUTIONS

Carlos Eduardo Costa: Conceptualization, data curation, formal analysis, methodology, project administration, resources, software, supervision, writing original draft, manuscript review and editing.

Karina Pinheiro da Silva: Conceptualization, data curation, formal analysis, investigation, methodology, resources, writing original draft.

André Connor de Méo Luiz: Writing original draft.

André Marques Choinski: Writing original draft.

Kennon A. Lattal: Writing original draft, manuscript review and editing.

## CONFLICT OF INTEREST STATEMENT

No conflict of interest.

## ETHICS APPROVAL

All experiments presented in this manuscript were conducted under a protocol approved by the Human Research Ethics Committee at Universidade Estadual de Londrina, Londrina‐PR, Brazil (Process number: CAAE: 55235516.5.0000.0093, Protocol 1.537.824/2016).

## Data Availability

The data are available on request from the first and second authors.
